# Comparison of diagnostic performances of ten different immunoassays detecting anti-CCHFV IgM and IgG antibodies from acute to subsided phases of Crimean-Congo hemorrhagic fever

**DOI:** 10.1371/journal.pntd.0009280

**Published:** 2021-03-15

**Authors:** Petra Emmerich, Ronald von Possel, Christina Deschermeier, Salih Ahmeti, Lindita Berisha, Bahrije Halili, Xhevat Jakupi, Kurtesh Sherifi, Claudia Messing, Viola Borchardt-Lohölter

**Affiliations:** 1 Department of Virology, Bernhard Nocht Institute for Tropical Medicine, WHO Collaborating Centre for Arbovirus and Hemorrhagic Fever Reference and Research, Hamburg, Germany; 2 University of Rostock, Rostock, Germany; 3 Diagnostics Development Laboratory, Bernhard Nocht Institute for Tropical Medicine, Hamburg, Germany; 4 National Institute for Public Health of Kosova, Prishtina, Kosovo; 5 University Clinical Center of Kosovo, Infectious Diseases Clinic, Prishtina, Kosovo; 6 Faculty of Agriculture and Veterinary Medicine, University of Prishtina “Hasan Prishtina”, Prishtina, Kosovo; 7 Institute for Experimental Immunology, affiliated with EUROIMMUN Medizinische Labordiagnostika AG, Luebeck, Germany; University of Surrey, UNITED KINGDOM

## Abstract

Crimean-Congo Hemorrhagic Fever Virus (CCHFV) is a geographically widespread tick-borne arbovirus that has been recognized by the WHO as an emerging pathogen needing urgent attention to ensure preparedness for potential outbreaks. Therefore, availability of accurate diagnostic tools for identification of acute cases is necessary.

A panel comprising 121 sequential serum samples collected during acute, convalescent and subsided phase of PCR-proven CCHFV infection from 16 Kosovar patients was used to assess sensitivity. Serum samples from 60 healthy Kosovar blood donors were used to assess specificity. All samples were tested with two IgM/IgG immunofluorescence assays (IFA) from BNITM, the CCHFV Mosaic 2 IgG and IgM indirect immunofluorescence tests (IIFT) from EUROIMMUN, two BlackBox ELISAs for the detection of CCHFV-specific IgM and IgG antibodies (BNITM), two Anti-CCHFV ELISAs IgM and IgG from EUROIMMUN using recombinant structural proteins of CCHFV antigens, and two ELISAs from Vector-Best (IgM: μ-capture ELISA, IgG: indirect ELISA using immobilized CCHFV antigen). Diagnostic performances were compared between methods using sensitivity, specificity, concordance and degree of agreement with particular focus on the phase of the infection.

In early and convalescent phases of infection, the sensitivities for detecting specific IgG antibodies differed for the ELISA test. The BlackBox IgG ELISA yielded the highest, followed by the EUROIMMUN IgG ELISA and finally the VectorBest IgG ELISA with the lowest sensitivities. In the subsided phase, the VectorBest IgM ELISA detected a high rate of samples that were positive for anti-CCHFV IgM antibodies. Both test systems based on immunofluorescence showed an identical sensitivity for detection of anti-CCHFV IgM antibodies in acute and convalescent phases of infection.

Available serological test systems detect anti-CCHFV IgM and IgG antibodies accurately, but their diagnostic performances vary with respect to the phase of the infection.

## Author summary

The Crimean-Congo Hemorrhagic Fever Virus (CCHFV) is the geographically most widespread tick-borne arbovirus and it is endemic in several countries. Because of its high case fatality rates, its potential for nosocomial outbreaks and the difficulties in treatment and prevention, CCHFV has been included in the WHO’s R&D Blueprint for Action to Prevent Epidemics. For fast implementation of infection control measures in CCHFV-affected countries, reliable diagnosis utilizing qualified molecular and serological tests is essential.

Here we compared diagnostic performances of ten serological tests based on detection of IgM and IgG antibodies against CCHFV using samples from CCHFV patients and samples from healthy blood donors collected in Kosovo. The analyses focused on the phase of infection, because IgM and IgG tests have differing intended purposes. We concluded that the tests are suitable for accurate detection of anti-CCHFV IgM and IgG antibodies, but varied in their respective diagnostic performances with respect to the phase of the infection. IgM tests are mainly intended to support the diagnosis of acute infections and performed well in the early and convalescent phases of infection. IgG tests become relevant at later stages of disease progression and for disease surveillance and reached highest sensitivities in the subsided phase of infection.

## Introduction

Crimean-Congo hemorrhagic fever orthonairovirus (CCHFV, family: *Nairoviridae*, genus: *Orthonairovirus*) is a tick-borne arbovirus that causes outbreaks of Crimean-Congo hemorrhagic fever (CCHF) [1,2]. CCHFV has the second widest geographical distribution of the arboviruses [2] and is endemic in Africa, the Balkans, the Middle East and Asia. Autochthonous cases have been observed in Spain [3]. CCHF occurs in recognized geographic foci and according to a regular seasonal pattern with a maximum of 1000 cases a year per country [4].

CCHFV is maintained in vertical and horizontal transmission cycles involving ticks and a variety of wild and domestic vertebrates, which themselves remain asymptomatic [1]. *Hyalomma* ticks are the principal source of human infection, although CCHFV can also be transmitted from contact with blood of infected animals or from person to person [5]. Infection with CCHFV occurs most frequently among agricultural workers. Slaughterhouse workers and medical personnel are also at risk of CCHFV infection, although to a lesser extent [1]. CCHF is the most important tick-borne viral disease of humans because it is associated with a case fatality rate of up to 40% [1,5,6].

The incubation period following a tick bite is usually one to three days or five to six days following contact with blood or tissues, respectively. The initial symptoms of the prehemorrhagic period (one to seven days) resemble those of other infectious syndromes with fever, headache, myalgia and gastrointestinal symptoms. Following the prehemorrhagic period, some cases develop a severe hemorrhagic disease, sometimes with major bleeding in and from the mucous membranes and the skin. The hemorrhagic period lasts for two to three days [2]. Some CCHF patients might develop a mild, nonspecific febrile illness and may not be recognized as being infected with CCHFV. Viral hemorrhagic fevers are clinically difficult to diagnose because the symptoms of CCHF are very similar to infection with Ebola and Marburg viruses, Lassa virus, Rift Valley fever virus, dengue virus and yellow fever virus [7]. CCHF may be diagnosed by direct virus detection during the first days after the onset of symptoms (dpso) by nucleic acid amplification tests and antigen detection, and from the second week after infection by serological testing for IgM antibodies for example by enzyme immunoassays (ELISA) and immunofluorescence testing (IFT) [8]. Both ELISA and IFT can be used to determine the serum titer via antibody titration. Patient management is mainly based on supportive care, because specific drugs or a licensed vaccine are not available [8–10]. In fatal cases, death generally occurs in the acute phase of up to 14 dpso as a result of hemorrhage, multi-organ failure and shock [1]. The convalescence phase follows the acute phase and usually lasts about ten days [2]. For patients who survived CCHF, the subsided phase starts a month after onset of symptoms and the recovery might take up to a year [11].

CCHFV infection can be confirmed by several laboratory tests: reverse transcriptase polymerase chain reaction (RT-PCR), virus isolation by cell culture, enzyme-linked immunosorbent assay (ELISA) for antibody or antigen detection, and serum neutralization. In patients with suspected CCHF, laboratory diagnosis can be made during the acute phase of the disease by positive results from antigen capture ELISA, RT-PCR and virus isolation. Quantitative RT-PCR has become the preferred method for fast, exact diagnosis [12]. Viral isolation is possible for CCHF diagnosis, but the number of laboratories that can perform this technique is limited because it has to be done, at least in non-endemic countries, in high-containment biosafety level 4 facilities [13]. One drawback of direct virus detection tests is their very short diagnostic window. PCR results may be negative if the sample had been taken later than nine days after symptom onset [8]. Serological testing has a longer diagnostic window starting from the hemorrhagic period of CCHF until recovery [8].

Detection of anti-CCHFV IgM antibodies is possible from approximately one week after the onset of disease until four months past infection [1,2,9]. During the course of CCHF infection, IgM antibodies are rapidly followed by IgG responses [14]. Anti-CCHFV IgG antibodies remain detectable for at least five years [15].

An acute phase CCHFV infection can be serologically confirmed by detecting anti-CCHFV IgM antibodies or specific IgM and IgG antibodies at the same time in an initial serum sample [2]. Recent or current infection can be confirmed by seroconversion or an at least fourfold increase in IgM antibody titre in paired serum samples [2]. High levels of specific IgM demonstrate recent infection, but they do not prove that the symptoms are caused by CCHFV. In areas where CCHFV is endemic, seropositivity may reflect recent infection with CCHFV with limited symptoms. Similar symptoms could be caused by other diseases e.g. Q fever, malaria, dengue [8]. The diagnostic value of seropositivity therefore depends on the prevalence of hemorrhagic fever viruses in the respective area of residence. In regions where CCHF is not endemic, positive IgM results are to be considered together with recent travel history of the patient [8]. Presence of anti-CCHFV IgG antibodies alone cannot confirm an ongoing infection with CCHFV. An antibody response is rarely detectable in fatal cases and is therefore an indicator for favorable disease outcome [11,16].

Currently available serological tests are highly specific and show no cross-reactivities [8]. However, their sensitivities are influenced by country of origin of the patients, potentially reflecting antigenic variation among CCHFV [17] and vary with respect to phase of the disease [18].

Because of its endemic potential, its high case fatality ratio, its potential for nosocomial outbreaks and the difficulties in treatment and prevention, CCHF constitutes a threat to public health services, and has been included in the World Health Organization’s Research and Development Blueprint for Action to Prevent Epidemics [6]. For prompt implementation of appropriate precautions and infection control measures to prevent the spread of CCHF, rapid and reliable diagnosis is essential [9]. One of the Blueprint’s strategic goals is to make affordable, qualified serology tests accessible to laboratories in CCHF-affected countries by 2020 [4]. Qualification of commercial IgM and IgG serological tests using panels of well-characterized clinical samples that cover the main circulating CCHFV strains is one milestone on the roadmap for 2020 [4]. Such serological tests will also be used for epidemiology and surveillance during outbreaks as well as for evaluation of vaccine immunogenicity and durability [4].

In this study, sequential serum samples from patients with confirmed CCHFV infection and single serum samples from healthy blood donors were collected in Kosovo between 2013 and 2016. Diagnostic performances of various commercial serological test systems (six enzyme-linked immunosorbent assays (ELISAs), four indirect immunofluorescence tests (IIFTs) for detection of IgM and IgG antibodies) were evaluated and compared. Particular focus was on the performance in different phases of the infection, because the tests based on anti-CCHFV IgM and IgG antibodies have differing intended purposes. IgM tests are mainly intended to support the diagnosis of acute infections, whereas IgG tests become relevant at later stages of disease progression as well as for epidemiology and disease surveillance.

## Material and methods

### Ethics statement

The study complies with the Declaration of Helsinki. Written informed consent was obtained from all individuals before enrollment. Data privacy protection was guaranteed by anonymization of serum samples. Collection of serum samples was approved by the Local Ethics Committee of the University of Prishtina “Hasan Prishtina”.

### Human sera

A serum panel comprising 121 sequential samples (25 samples from acute phase: 6–14 days post symptom onset (dpso), 13 samples from convalescent phase: 15–27 dpso, 83 samples from subsided phase: after 28 dpso) collected in Kosovo from 16 patients (13 males, mean age: 38.3 years, range: 10–63 years, age and gender are unknown for one patient) with CCHFV infection was used to assess sensitivity (Tables 1 and S5). All patients showed symptoms corresponding to CCHF and infection with CCHFV has been confirmed by RT-PCR (RealStar CCHFV RT PCR Kit, Altona Diagnostics) in a serum sample collected directly after admission to the hospital. Furthermore, all initial samples were positive for IgG antibodies against CCHFV by immunofluorescence assays (IFA) tested in the WHO Collaborating Centre for Arbovirus and Hemorrhagic Fever Reference and Research (Hamburg, Germany). Serum samples from 60 healthy Kosovar blood donors (49 males, mean age: 35 years, range: 20–60 years) were used to assess specificity (S6 Table).

**Table 1:** Grouping of 121 serum samples in three phases of CCHFV infection.

Phase of infection	N serum samples per phase	Days post symptom onset		
		grouping	mean ± sd	range
**acute**	25	6–14	9.8 ± 2.3	[6,14]
**convalescent**	13	15–27	17.8 ± 2.4	[15,21]
**subsided**	83	≥ 28	1219 ± 675.2	[28,2222]

Sd: standard deviation.

### Serological test systems

All serum samples were tested for presence of anti-CCHFV antibodies with the following ten serological methods:

- the IgM/IgG IFA from Bernhard Nocht Institute for Tropical Medicine (BNITM, Hamburg, Germany, abbreviated: BNITM IgM/IgG IFA),
- the Crimean-Congo Fever Virus Mosaic 2 (IgM or IgG) indirect immunofluorescence tests (IIFT) from EUROIMMUN (Lübeck, Germany) detecting IgM or IgG antibodies against the CCHFV glycoproteins (GPC–glycoprotein precursor) and CCHFV nucleocapsid protein (N) (abbreviated: EI IgM/IgG IIFT),
- the μ-capture ELISA for the detection of CCHFV-specific IgM antibodies (BLACKBOX CCHFV IgM, abbreviated: BB IgM ELISA) and IgG immune complex ELISA for the detection of CCHFV-specific IgG antibodies (BLACKBOX CCHFV IgG, abbreviated: BB IgG ELISA) from BNITM [18],
- the newly developed Anti-Crimean-Congo Hemorrhagic Fever Virus (CCHFV) ELISA IgM and Anti-Crimean-Congo Hemorrhagic Fever Virus (CCHFV) ELISA IgG (abbreviated: EI IgM ELISA and EI IgG ELISA) from EUROIMMUN, using recombinant structural proteins of CCHFV as antigens, and
- the VectoCrimean-CHF-IgM/IgG ELISAs from Vector-Best (Koltsovo, Russia) (IgM: μ-capture ELISA, IgG: indirect ELISA using immobilized CCHFV antigen (abbreviated: VB IgM ELISA and VB IgG ELISA).

All serological tests were performed and evaluated according to the manufacturer’s instructions.

### Statistical analysis

Qualitative results obtained for each diagnostic tool were compared via several 2×2 contingency tables. Diagnostic performances of the test systems were quantified concerning sensitivity and specificity. Borderline results were defined according to the respective test’s instruction and evaluated as positive. For the BNITM IFA tests, a titer of 1:20 was considered as borderline. For the EI IIFT IgM and IgG, a titer of 1:10 and 1:100, respectively, was considered as borderline.

Agreement between serological tests was assessed using McNemar test [19] with χ^2^ approximation without Yates’s correction for continuity. To correct for multiple testing, Bonferroni correction was applied. As measures of effect size, McNemar odds ratios and their 95% confidence intervals based on Wilsons’s score interval without Yates’s continuity correction are reported (S1 Table). To circumvent the zero-cell problem, Haldane-Anscombe correction was applied when necessary. Tests for IgG and IgM were considered to be independent.

Based on the contingency table for each comparison of two tests, concordance was calculated (percentage agreement = samples with identical outcome in both respective methods divided by number of samples *100).

For each comparison, the degree of agreement (Cohen’s kappa) was calculated based on contingency tables containing data from both serum panels. Degree of agreement was interpreted as follows: κ≤0.2 = slight, 0.21<κ≤0.4 = fair, 0.41<κ≤0.6 = moderate, 0.61<κ≤0.8 = substantial, κ>0.8 = almost perfect, κ = 1 = perfect.

## Results

### Comparison of test systems detecting specific IgM antibodies

The positivity rate for detection of anti-CCHFV IgM antibodies in the blood donor panel was 0% (specificity = 100%, 95% CI: 94.0% to 100%) in all test systems except for the EI ELISA, which had a specificity of 98.3% (95% CI: 91.1% to 100%, Table 2).

**Table 2:** Diagnostic specificity of 60 serum samples from healthy Kosovar blood donors. Comparison of results of BNITM IgM IFA, EUROIMMUN IgM IIFT, BLACKBOX IgM ELISA, EUROIMMUN IgM ELISA and VectorBest IgM ELISA for detection of anti-CCHFV antibodies of class IgM and IgG.

Antibody class	Negative for anti-CCHFV antibodies in									
	BNITM IFA		EI IIFT		BB ELISA		EI ELISA		VB ELISA	
	n	specificity	n	specificity	n	specificity	n	specificity	n	specificity
IgM	60	100%	60	100%	60	100%	59	98.3%	60	100%
IgG	60	100%	57	95%	60	100%	59	98.3%	60	100%

Results of the McNemar test between all IgM test systems revealed significant differences between BNITM IFA versus BB ELISA, BNITM IFA versus VB ELISA, EI IIFT versus BB ELISA, EI IIFT versus VB ELISA and all ELISAs (S1 Table).

Anti-CCHFV IgM antibodies were reliably detected by all test systems (sensitivities: 100%, 95% CI: 86.3% to 100%) during early and convalescent phases of the infection (Table 3). In the subsided phase of infection, the sensitivities dropped to 21.7–80.7% (Table 3).

**Table 3:** Diagnostic sensitivity of paired CCHF patient samples in IgM tests. Comparison of results of BNITM IgM IFA, EUROIMMUN IgM IIFT, BLACKBOX IgM ELISA, EUROIMMUN IgM ELISA and VectorBest IgM ELISA for detection of anti-CCHFV IgM antibodies with respect to phase of infection.

Phase of infection	N samples	Positive for anti-CCHFV IgM antibodies in									
		BNITM IFA		EI IIFT		BB ELISA		EI ELISA		VB ELISA	
		n	sensitivity	n	sensitivity	n	sensitivity	n	sensitivity	n	sensitivity
**acute**	25	25	100%	25	100%	25	100%	25	100%	25	100%
**convalescent**	13	13	100%	13	100%	13	100%	13	100%	13	100%
**subsided**	83	20	24.1%	18	21.7%	55	66.3%	21	25.3%	67	80.7%
**all phases**	121	58	47.9%	56	46.3%	93	76.9%	59	48.8%	105	86.8%

The BB IgM ELISA detected more IgM antibodies in samples collected in the subsided phase than the EI IgM ELISA (sensitivity BB IgM ELISA: 66.3% (95% CI: 55.1% to 76.3%); sensitivity EI IgM ELISA: 25.3% (95% CI: 16.4% to 36%); Table 3).

The VB IgM ELISA detected more IgM antibodies in samples collected in the subsided phase than the EI IgM ELISA (sensitivity VB IgM ELISA: 80.7% (95% CI: 70.6% to 88.6%); sensitivity EI IgM ELISA: 25.3% (95% CI: 16.4% to 36%); Table 3).

While concordance between test systems was 100% in samples from acute and convalescent phases of infection, in samples from the subsided phase the concordance ranged from 38.6 to 85.5 percent positive agreement (S2 Table). BNITM IFA and VB ELISA showed a low concordance of 41% in samples from the subsided phase of infection (S2 Table). EI IIFT and VB ELISA showed a low concordance of 38.6% in the subsided phase of infection (S2 Table).

When taking both serum panels into account, the degrees of agreement measured by Cohen’s kappa ranged from almost perfect to perfect during early and convalescent phases of the infection (Table 4). During the subsided phase of the infection, the degrees of agreement ranged from fair to moderate, with the exception of the comparison between BB ELISA and VB ELISA showing a κ of 0.83 (Table 4). The lowest degree of agreement was observed in the subsided phase for EI IIFT and VB ELISA (κ = 0.25).

**Table 4:** Degree of agreement between IgM test results. Degrees of agreement between results of BNITM IgM IFA, EUROIMMUN IgM IIFT, BLACKBOX IgM ELISA, EUROIMMUN IgM ELISA and VectorBest IgM ELISA results based on serum samples from blood donors and CCHF patients with respect to phase of infection. Cohen’s κ is listed for every comparison. Dark green cells correspond to an almost perfect and perfect agreement, bright green cells correspond to a substantial agreement, pale green cells correspond to a moderate agreement and white cells correspond to a fair agreement.

Phase of infection	BNITM IFA vs. EI IIFT	BNITM IFA vs. BB ELISA	BNITM IFA vs. EI ELISA	BNITM IFA vs. VB ELISA	EI IIFT vs. BB ELISA	EI IIFT vs. EI ELISA	EI IIFT vs. VB ELISA	BB ELISA vs. EI ELISA	BB ELISA vs. VB ELISA	EI ELISA vs. VB ELISA
**acute**	1	1	0.97	1	1	0.97	1	0.97	1	0.97
**convalescent**	1	1	0.95	1	1	0.95	1	0.95	1	0.95
**subsided**	0.51	0.35	0.44	0.28	0.31	0.30	0.25	0.38	0.83	0.32
**all phases**	0.79	0.57	0.75	0.49	0.55	0.69	0.47	0.59	0.87	0.51

**Fig 1:**
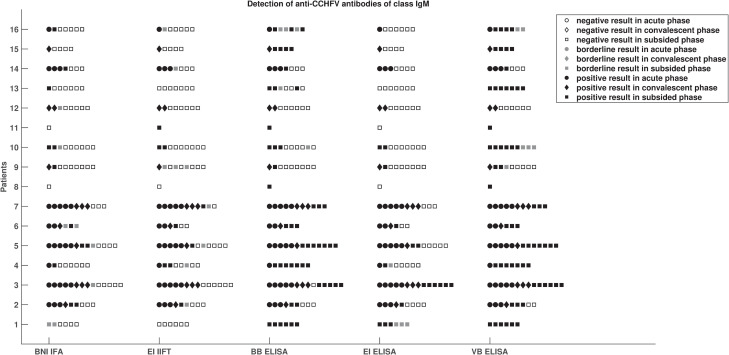
Comparison of IgM test results. Comparison of results from five serological test systems for the detection of anti-CCHFV antibodies of class IgM with respect to phase of infection of 16 patients with CCHFV infection.

The most striking observation to emerge from the method comparison of IgM test systems is that the VB IgM ELISA showed an unusually high rate of samples positive for anti-CCHFV IgM antibodies in the subsided phase of infection (Fig 1). Both test systems based on immunofluorescence performed identically in the acute and convalescent phase of infection (Fig 1). S1 Fig depicts IgM antibody kinetics assessed by each method based samples from one exemplary patient across acute, convalescent and subsided phases of infection.

### Comparison of test systems detecting specific IgG antibodies

The positivity rate for detection of anti-CCHFV IgG antibodies in the blood donor panel was 0% (specificity = 100%, 95% CI: 94.0% to 100%) in all test systems except the EI IIFT and EI ELISA, which showed specificities of 95% (95% CI: 86.1% to 99%) and 98.3% (95% CI: 91.6% to 100%), respectively (Table 2).

Results of the McNemar test between all IgG test systems revealed significant differences between BNITM IFA versus EI ELISA, BNITM IFA versus VB ELISA, EI IIFT versus EI ELISA, EI IIFT versus VB ELISA, BB ELISA versus VB ELISA and EI ELISA versus VB ELISA (S1 Table).

In the acute phase of infection, the test systems reached sensitivities between 100% and 12% (Table 5). In the convalescent phase of infection, the test systems reached sensitivities between 100% and 92.3%, except the VB ELISA, which showed a sensitivity of 69.2% (Table 5). All IgG test systems reliably detected specific CCHFV IgG antibodies (sensitivities: 98.8% to 100%) in the subsided phase of the infection (Table 5).

**Table 5:** Diagnostic sensitivity of paired CCHF patient samples in IgG tests. Comparison of results of BNITM IgG IFA, EUROIMMUN IgG IIFT, BLACKBOX IgG ELISA, EUROIMMUN IgG ELISA and VectorBest IgG ELISA for detection of anti-CCHFV IgG antibodies with respect to phase of infection.

Phase of infection	N samples	Positive for anti-CCHFV IgG antibodies in									
		BNITM IFA		EI IIFT		BB ELISA		EI ELISA		VB ELISA	
		n	sensitivity	n	sensitivity	n	sensitivity	n	sensitivity	n	sensitivity
**acute**	25	25	100%	22	88.0%	20	80.0%	13	52.0%	3	12.0%
**convalescent**	13	13	100%	13	100%	13	100%	12	92.3%	9	69.2%
**subsided**	83	83	100%	83	100%	82	98.8%	82	98.8%	82	98.8%
**all phases**	121	121	100%	118	97.5%	115	95%	107	88.4%	94	77.7%

The BB IgG ELISA was more sensitive than the VB IgG ELISA in detecting anti-CCHFV IgG antibodies in samples collected in acute and convalescent phases (acute phase: sensitivity BB IgG ELISA: 80% (95% CI: 59.3% to 93.2%); sensitivity VB IgG ELISA: 12% (95% CI: 2.6% to 31.2%); convalescent phase: sensitivity BB IgG ELISA: 100% (95% CI: 75.3% to 100%); sensitivity VB IgG ELISA: 69.2% (95% CI: 38.6% to 90.9%); Table 5).

The EI IgG ELISA was more sensitive than the VB IgG ELISA in detecting anti-CCHFV IgG antibodies in samples collected in acute and convalescent phases (acute phase: sensitivity EI IgG ELISA: 52% (95% CI: 31.3% to 72.2%); sensitivity VB IgG ELISA: 12% (95% CI: 2.6% to 31.2%); convalescent phase: sensitivity EI IgG ELISA: 92.3% (95% CI: 64% to 99.8%); sensitivity VB IgG ELISA: 69.2% (95% CI: 38.6% to 90.9%); Table 5).

While concordance between test systems ranged between 12% and 92% in the acute phase, it increased to a range from 66.7% to 100% in the convalescent phase of infection (S3 Table). In the subsided phase the concordance was between 98.8% and 100% (S3 Table). BNITM IFA and EI IIFT showed a concordance of 100% in both convalescent and subsided phases of infection (S3 Table). The three ELISA tests showed a concordance of 100% in the subsided phase of infection (S3 Table). BNITM IFA and VB ELISA showed a low concordance of 12% in the acute phase of infection (S3 Table). EI IIFT and VB ELISA showed a low concordance of 24% in the acute phase of infection (S3 Table).

When taking both serum panels into account, the agreement between IgG tests was almost perfect during the subsided phase of the infection (Table 6). During the acute and convalescent phases of the infection, the degrees of agreement ranged from slight to perfect (Table 6). The lowest degrees of agreement (κ = 0.16) were observed in the acute phase for BNITM IFA versus VB ELISA as well as EI IIFT versus VB ELISA.

**Table 6:** Degree of agreement between results of IgG tests. Degrees of agreement between results of BNITM IgG IFA, EUROIMMUN IgG IIFT, BLACKBOX IgG ELISA, EUROIMMUN IgG ELISA and VectorBest IgG ELISA results based on serum samples from healthy blood donors and CCHFV patients with respect to phase of infection. Cohen’s κ is listed for every comparison. Dark green cells correspond to an almost perfect and perfect agreement, bright green cells correspond to a substantial agreement, pale green cells correspond to a moderate agreement and white cells correspond to a slight or fair agreement.

Phase of infection	BNITM IFA vs. EI IIFT	BNITM IFA vs. BB ELISA	BNITM IFA vs. EI ELISA	BNITM IFA vs. VB ELISA	EI IIFT vs. BB ELISA	EI IIFT vs. EI ELISA	EI IIFT vs. VB ELISA	BB ELISA vs. EI ELISA	BB ELISA vs. VB ELISA	EI ELISA vs. VB ELISA
**acute**	0.83	0.85	0.58	0.16	0.85	0.69	0.16	0.71	0.21	0.18
**convalescent**	0.87	1	0.91	0.79	0.86	0.92	0.64	0.91	0.79	0.79
**subsided**	0.96	0.99	0.97	0.99	0.94	0.97	0.94	0.97	0.99	0.97
**all phases**	0.93	0.93	0.82	0.70	0.93	0.86	0.70	0.90	0.77	0.82

**Fig 2:**
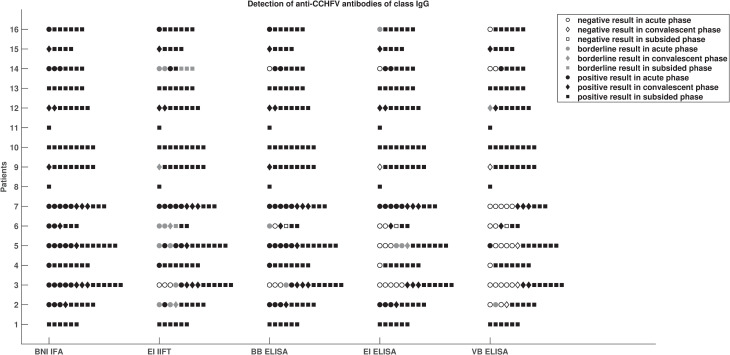
Comparison of IgG test results. Comparison of results from five serological test systems for the detection of anti-CCHFV antibodies of class IgG with respect to phase of infection of 16 patients with CCHFV infection.

The most striking observation to emerge from the method comparison of IgG test systems is that the EI IgG ELISA detected more samples positive for anti-CCHFV IgG antibodies in acute and convalescent phases of infection than the VB IgG ELISA (Fig 2). Furthermore, the diagnostic sensitivity of the EI IgG IIFT was very similar to that of the BNITM IFA, the only difference being that the EI IgG IIFT did not detect anti-CCHFV IgG antibodies in three acute phase samples from one patient (Fig 2). S2 Fig depicts IgG antibody kinetics assessed by each method based samples from one exemplary patient across acute, convalescent and subsided phases of infection.

## Discussion

### Summary

The aim of this study was to compare diagnostic performances of ten available test systems for detection of anti-CCHFV antibodies of classes IgM and IgG. Serum samples from patients with confirmed CCHFV infection and from healthy blood donors were tested with six ELISAs and four immunofluorescence tests. The samples were categorized according to the time of collection during the course of the infection, i.e. acute, convalescent or subsided phase. These phases were of specific interest, because of the differing intended purposes for IgM- and IgG tests. The test systems detected anti-CCHFV IgM and IgG antibodies accurately, but a variation of diagnostic performances was observed with regard to the phase of the infection.

### Detection of specific IgM antibodies with respect to phases of infection

An accurate early diagnosis of CCHF is of tremendous importance, because detection of an acute case of CCHF initiates strict infection control measures to prevent secondary nosocomial and community-level virus transmission. IgM tests are mainly intended for the support of the diagnosis of acute infections. Hence, diagnostic performances based on samples from the acute and convalescent phases of infection are of primary relevance to evaluate the usefulness of the test systems.

The positivity rate for detection of anti-CCHFV IgM antibodies in the blood donor panel was 0% (specificity = 100%) in all test systems with the exception of the EI ELISA, which had a specificity of 98.3%. All test systems detected anti-CCHFV IgM antibodies with maximal sensitivities during early and convalescent phases of infection in sera from patients with CCHF. When taking both serum panels into account, the agreement between test systems was almost perfect to perfect during early and convalescent phases of the infection but decreased during the subsided phase of infection (Table 4).

One unanticipated finding was that the VB IgM ELISA detected anti-CCHFV IgM antibodies in 67 samples collected in the subsided phase (up to 72 months post symptom onset), followed by the BB IgM ELISA (55 samples, up to 72 months post symptom onset) and finally the EI IgM ELISA, BNITM IgM IFA and EI IgM IIFT (21, 20 and 18 samples, and up to 60, 41, and 54 months post symptom onset, respectively). Of the 83 samples collected in the subsided phase, 74 samples were collected later than four months after onset of symptoms. These 74 samples were expected to test negative for anti-CCHFV IgM antibodies, because anti-CCHFV IgM antibodies disappear four months after onset of symptoms [2]. Instead, detection of anti-CCHFV IgM antibodies was expected in the nine remaining samples, which were collected earlier than four months after onset of symptoms. The detection of anti-CCHFV IgM antibodies in high shares in samples collected in the subsided phase might indicate false positive results and should be interpreted with caution. These positive results could also be due to cross-reactions, for example with Hantavirus, but the extent of cross-reactivities has not been investigated in the current study. Alternatively, a positive result for IgM in a sample collected in a presumed late phase of the infection could also be explained by an acute secondary infection with CCHFV. In this case, the positive result would be true. Taken together, the diagnostic implication of an IgM-positive sample collected in the subsided phase is ambiguous and necessitates verification to ensure appropriate patient management while avoiding needless infection control measures.

The observation that serum samples taken from CCHF patients more than one year after having overcome the disease tested weakly positive in the BB IgM ELISA was also reported earlier [18]. A clear differentiation between acute and subsided states of CCHF is possible by calculating the IgG/IgM ratio (defined as the quotient of the optical densities obtained when performing the BB IgG ELISA and the BB IgM ELISA, respectively), which is lower in the patient samples taken during the acute phase than in samples taken during the subsided phase [18]. It is recommended that a positive result by BB IgM ELISA be assessed by additional testing and/or clinical findings. Regarding positive results obtained by the EI IgM ELISA and the EI IgM IIFT, the manufacturer mentions that positive findings may also be related to polyclonal stimulation of the immune system or antibody persistence. According to the test instruction of the VB IgM ELISA, it is necessary to repeat analysis of serum to avoid false positive results caused by random, non-systemic errors during the analysis. In summary, the diagnostic performances of the IgM test systems are accurate when applied according to their respective intended purposes and IgM-positive results in samples collected in the late phase of CCHFV infection should be treated with caution.

### Detection of specific IgG antibodies with respect to phases of infection

Specific IgG antibodies are detectable by the end of the first week of illness and persist for years. Despite the relatively early appearance of anti-CCHFV IgG antibodies, IgG tests are mainly used for disease monitoring in addition to epidemiology studies and surveillance of potential outbreaks. Hence, diagnostic performances based on samples from the subsided phase of infection are of primary interest to evaluate the usefulness of the IgG test systems.

The specificity for detection of anti-CCHFV IgG antibodies in the blood donor panel was 100% in all test systems except the EI IIFT and EI ELISA, which showed specificities of 95% and 98.3%, respectively. In the subsided phase, all IgG test systems reliably detected anti-CCHFV IgG antibodies (sensitivities: 98.8% to 100%) and the concordance between test systems ranged from 98.8% to 100%. When taking both serum panels into account, the agreement between all IgG tests was almost perfect during the subsided phase of the infection (Table 6). It can therefore be concluded that all test systems perform well in detecting anti-CCHFV IgG antibodies late in the course of the disease, which is in accordance with their intended purpose.

However, when analyzing diagnostic performances of IgG tests in samples collected during the acute and convalescent phases of infection, differences were evident. The EI IgG IIFT showed high sensitivities in acute and convalescent phases (88% and 100%, respectively). The most interesting observation was that among the ELISA tests the BB IgG ELISA yielded the highest, followed by the EI IgG ELISA and finally the VB IgG ELISA with the lowest sensitivities (acute phase: 80% versus 52% versus 12%, convalescent phase: 100% versus 92.3% versus 69.2%). This observation reflects the fact that the BB and EI IgG ELISAs as well as the EI IgG IIFT provided the most accurate test results during early phases of CCHF, thereby exceeding the requirements of the intended purpose of IgG tests.

Tests that show a high sensitivity in detection of anti-CCHFV IgG antibodies in samples collected in early stages of the infection can support accurate serodiagnosis, e.g. in the event of an IgM borderline test result. Currently, recent infection is confirmed by seroconversion or an at least fourfold increase in IgM antibody titre in paired serum samples. With highly sensitive IgG tests at hand, an increase in IgG antibody titre could present an alternative to confirm a recent infection. Moreover, presence of anti-CCHFV IgG antibodies in the acute phase of infection can also serve as an indicator for convalescence and favorable disease outcome [11].

Notably, cross-reactivities have not been investigated here, but could have affected IgG-positive results. Our conclusion is that the analyzed IgG test systems comply well with the intended purpose of disease monitoring and seroprevalence studies.

### General differences between the test systems

When comparing diagnostic performances of several test systems, general differences in the technique and used antigens of the tests need to be taken into account. For example, results are influenced by differing antigens (S4 Table). While the BNITM IFA is performed on CCHFV-infected Vero-cells at presence of all viral antigens, in the EI IIFT tests, detection of antibodies against CCHFV is performed using recombinant proteins. This increases the diagnostic capability for the recognition of CCHFV-associated antibodies. It has been speculated that the very first IgG antibodies generated during acute CCHFV infection primarily recognize the virus envelope glycoprotein (CCHFV-GPC) instead of the CCHFV nucleoprotein (CCHFV-NP) [18].

The findings presented in the current study are limited by the number of included samples and further as a result of an uneven distribution of samples across the three phases of infection. The study included two patients from whom only one serum sample was available, whereas it was possible to collect between five and 14 samples from the other patients. Moreover, influences of country of origin could not be investigated in this study, because the included patients and blood donors were residents of the same geographical region. Finally, diagnostic tests are often evaluated using the strains most relevant to that region or outbreak [20]. Although serological assays are sensitive to antigenic variation, they are however generally less impacted by genetic variation [17]. This method comparison did not include an analysis of which CCHFV strain caused the infection.

## Conclusions

Infections with CCHFV have a high epidemic potential. To prevent their spread, fast implementation of appropriate infection control measures is essential. Several serological tests for the laboratory diagnosis of CCHFV infection are available commercially and their diagnostic performances have been compared in the current study. In conclusion, the available serological test systems proved themselves suitable for accurate detection of anti-CCHFV IgM and IgG antibodies, but varied in their respective diagnostic performances with respect to the phase of the infection. The IgM test systems performed well in the early and convalescent phases of infection, whereas the IgG test systems reached highest sensitivities in the subsided phase of infection. The results of this study give guidance on which type of serological test for detection of anti-CCHFV antibodies could be used in different phases of the infection and could provide urgently needed information for studies assessing the seroprevalence of against CCHFV antibodies in endemic areas and could also help to keep countries in danger of virus emergence under surveillance.

## Supporting information

S1 Fig Kinetics of anti-CCHFV IgM and IgG antibodies based on sequential serum samples from patient 7. Results of each serological testing method are visualized. Dashed and solid horizontal lines represent the cut-off ratios for borderline and positive results, respectively.
(EPS)

S2 Fig Kinetics of anti-CCHFV IgM and IgG antibodies based on sequential serum samples from patient 3. Results of each serological testing method are visualized. Dashed and solid horizontal lines represent the cut-off ratios for borderline and positive results, respectively.
(EPS)

S1 Table Statistical analysis of CCHF patient samples. Comparison of results of BNITM IFAs, EUROIMMUN IIFTs, BLACKBOX ELISAs, EUROIMMUN ELISAs and VectorBest ELISAs based on samples collected throughout the infection (n = 121). P-values (p), Odds Ratio (OR), and 95% confidence interval (CI) of McNemar test are listed for every comparison between two serological tests. P-values that are significant after correction for multiple comparisons are typeset in bold.
(XLSX)

S2 Table Concordance of paired CCHF patient samples in IgM tests. Comparison of BNITM IgM IFA, EUROIMMUN IgM IIFT, BLACKBOX IgM ELISA, EUROIMMUN IgM ELISA and VectorBest IgM ELISA results with respect to phase of infection. Number and percentage of positive agreement are listed for every comparison. The dataset comprises 121 sequential samples (25 samples from acute phase, 13 samples from convalescent phase, 83 samples from subsided phase) collected from 16 patients with CCHF.
(XLSX)

S3 Table Concordance of paired CCHF patient samples in IgG tests. Comparison of BNITM IgG IFA, EUROIMMUN IgG IIFT, BLACKBOX IgG ELISA, EUROIMMUN IgG ELISA and VectorBest IgG ELISA results with respect to phase of infection. Number and percentage of positive agreement are listed for every comparison. The dataset comprises 121 sequential samples (25 samples from acute phase, 13 samples from convalescent phase, 83 samples from subsided phase) collected from 16 patients with CCHF.
(XLSX)

S4 Table Antigens used in the different test systems. The BNITM IFA is performed on CCHFV-infected Vero-cells at presence of all viral antigens. In the EI IIFT tests, detection of antibodies against CCHFV is performed using recombinant proteins. The two BB ELISAs [18] employ recombinant CCHFV-NP as antigen. The CCHFV-NP was recombinantly expressed in E. coli, purified and directly labelled with horseradish peroxidase. For the BB IgM ELISA, plates are coated with anti-human IgM antibodies. The BB IgG ELISA employs the IgG immune complex binding principle. In this type of ELISA, the recombinantly produced tandem immunoglobulin-like domain of the human FcγR CD32 is used as a capture molecule to bind immune complexes formed between pathogen-specific IgG antibodies and either native or recombinant viral antigens [21]. The wells of the EI ELISAs are coated with a mixture of the recombinant structural proteins of the Crimean-Congo hemorrhagic fever virus. The VB IgM test is a μ-capture ELISA. The VB IgG test is an indirect ELISA using immobilized CCHFV antigen. VectorBest does not provide information about the used antigen.
(XLSX)

S5 Table Dataset of CCHFV patients. (XLSX)

S6 Table Dataset of healthy blood donors. (XLSX)
